# Non-Invasive Challenge Response Authentication for Voice Transactions with Smart Home Behavior

**DOI:** 10.3390/s20226563

**Published:** 2020-11-17

**Authors:** Victor Hayashi, Wilson Ruggiero

**Affiliations:** Polytechnic School, University of São Paulo, São Paulo 05508-010, Brazil; wilson@larc.usp.br

**Keywords:** smart home, machine learning, authentication, simulation, test bed

## Abstract

Smart speakers, such as Alexa and Google Home, support daily activities in smart home environments. Even though voice commands enable friction-less interactions, existing financial transaction authorization mechanisms hinder usability. A non-invasive authorization by leveraging presence and light sensors’ data is proposed in order to replace invasive procedure through smartphone notification. The Coloured Petri Net model was created for synthetic data generation, and one month data were collected in test bed with real users. Random Forest machine learning models were used for smart home behavior information retrieval. The LSTM prediction model was evaluated while using test bed data, and an open dataset from CASAS. The proposed authorization mechanism is based on Physical Unclonable Function usage as a random number generator seed in a Challenge Response protocol. The simulations indicate that the proposed scheme with specialized autonomous device could halve the total response time for low value financial transactions triggered by voice, from 7.3 to 3.5 s in a non-invasive manner, maintaining authorization security.

## 1. Introduction

Smart assistants are making natural interactions through voice commands that are accessible in smart home environments. Google Home installed base in smartphones and smart speakers exceeds one-billion devices [1], while Amazon Alexa is deployed in more than 100 million smart speaker devices [2]. Actually, 20% of U.S. adults reported having a smart speaker in 2018 [3].

Voice interactions provide pervasive services by integration with Internet of Things (IoT) devices tgar are present in smart homes. Ericsson estimates 18 billion of these connected devices by 2022 [4]. These everyday objects with sensing, processing, and actuation capabilities communicate with each other to offer convenient services to users in a timely manner [5].

Transactions that are triggered by voice in these environments could range from P2P (person to person) money transfers, utility bill [6], and food delivery [7] payments. Axis Bank Alexa Skill [8] makes it possible for users to access their balance, get credit card bill, query transaction history, and even block cards; American Express Alexa Skill [9] enables users to access account information, and make payments; and, Capital One Alexa Skill [10] lets users check their balance, track spending, and pay bills.

Existing authorization solutions for these transactions are based upon invasive user authentication through smartphone notification [11], which hinders usability by increased friction in these interactions. Requiring confirmation by an interface other than speech makes users frustrated when interacting with personal assistants [12]; Alexa thus provided Alexa PIN, a four-digit PIN required for each user interaction with American Express Skill [13]. SMS code and spoken four-digit codes are commonly used along with voice recognition [12]. As far as the authors are concerned, security and usability trade off in smart speakers financial transactions still remains an open question.

In the case of Axis Bank Skill, its Terms and Conditions state that the Bank is not held responsible for the possible loss that is incurred for Alexa PIN misuse [14]. Capital One Alexa Skill Terms and Conditions make it clear that voice command is not used for authentication, and that Amazon could have access over conversations performed [15]. Private conversations have already been sent by Alexa to contacts without user permission [16], and privacy claims have also been made [17].

Therefore, the research question is: how to authorize low-value (up to $50) financial transactions that are triggered by voice in smart home environments, in an non-invasive manner (i.e., not requiring additional user interaction other than voice command)?

Our contribution integrates public access to software and data sets, which can be found under:

- Public GIT repository [18]: software modules for synthetic data generation (coloured petri nets), data processing and algorithms for classification and prediction in single user and multi-user scenarios;
- Database [19]: public access to 2019 smart home dataset of three months from Brazilian smart home test bed.
- Data logger [20]: open data logger firmware module to accelerate offline data acquisition using ESP8266 IoT module through easy integration to Arduino projects and web-based interface.

This paper is organized, as follows: literature review on smart homes is presented in Section 2 and an overview of proposed authentication mechanism is presented in Section 3. The method for evaluating the proposed solution through simulation and test bed data is described in Section 4, and the quantitative results of some machine learning algorithms leveraging smart home data can be found in Section 5. Proposed authentication that is based on challenge response protocol is detailed in Section 6 with autonomous device specification, enrollment, and protocol descriptions. A comparison of results with related work is presented in Section 7. Section 8 presents final considerations regarding the results presented and directions for future work.

## 2. Related Work

Given the increased mobility of IoT devices [21,22], existing authentication mechanisms are less effective [22]. Energy and computing power of constrained IoT devices limit asymmetric cryptography methods use in a smart home scenario, as these methods allow for secure mechanisms implementation, but at high computational expense [23].

Even probable solutions, such as voice recognition, should not be considered a panacea, as stated in [24]. The feasibility of inaudible audio attacks in speech recognition systems, such as Siri and Alexa, was proven in [25]. Speaker recognition systems based on deep neural networks are also vulnerable to adversarial attacks, as shown by the high attack success rate of over 90% [26].

Novel mechanisms that combine diverse authentication factors can be used as an alternative to static passwords. Such multi-factor authentication solutions could lessen authentication procedures for the trusted user profiles [27] and they could be a robust alternative to the reliance upon unique authentication factor such as voice recognition.

Another challenge is smart speaker privacy: no support to voice recognition by default in Alexa [24], and always on, always listening aspects raises user concerns [28]. Weak payment authentication is also a security issue: insider unauthorized orders (e.g., a kid making an order of $300 with its parent account) have already happened [29].

An emerging paradigm is context validation, which aims to verify the presence of a user by a secondary channel in an authentication protocol. Some examples are based on Wi-Fi and Bluetooth communication channels [30].

Real-time security mechanisms could utilize smart home contextual information to authorize requests, without requiring additional user intervention [22]. Whereas, users demand context-aware data access control policies (e.g., who is at home and where the request comes from) [31], smart speaker user privacy perception is still at an early stage (e.g., voice recognition services are available, but are not broadly used [32]). A mechanism that is based on contextual rules for IP camera streaming authorization is proposed for monitoring babysitters and children in smart homes [33]. Smart Home security could be composed of sensing, detection, and response mechanisms, as presented in a survey that is found in the literature [34].

A study intended to make proximity-based payments resilient to replay attacks proposed using environment data that were collected by mobile devices [35]. Another work used total response time metric in order to evaluate usability in financial transaction scenarios [36], and a study regarding real-time inference in edge devices evaluated response time to consider user perspective advantages of the proposed approach [37]. An exploratory study regarding user recognition based on Alexa Skills usage is presented in [38].

Some studies propose architectures for smart home data-collection systems aiming to be easily installed in real deployments. The E-care@home system [5] provides a solution for elderly monitoring with wearable and stationary sensors. It also includes a person counting module that is intended to facilitate data collection in multi-user scenarios, with the benefit of being less intrusive than vision-based sensors.

The CASAS project [39] from Washington State University provides an easy to install smart home data collection module with pre-labeled sensors indicating the intended location. Temperature, motion, and open/closed sensors are provided for event-oriented data collection. The kit aims to create smart home datasets for activity classification, and includes a web interface to help researchers visualize the activities of daily living during system calibration.

The idea of applying algorithms to smart home data to obtain insights about residents is not new. A perspective in activity recognition could be considering vision and sensor-based monitoring as the main approaches for monitoring. Sensor-based approaches can be divided into data- and knowledge-driven approaches. In a data-driven approach, data mining and machine learning techniques are employed in order to learn human behaviour, and some models that are found in the literature are SVM, hidden Markov, and Bayesian networks models. Knowledge-driven approaches use domain knowledge in order to overcome the drawback of manual activity labeling (e.g., through the use of ontologies) [40,41]. Daily activity recognition leveraging smart home data is presented in [34].

Activity recognition research can be divided into five categories, according to the monitoring technology: video cameras, criticized for privacy exposure and cost; sound recognition; body-worn sensors; pressure sensors placed on the floor; and, ambient sensors, based on sensor events. The last approach has the benefits of privacy protection, resident freedom from carrying extra devices, but the drawback of the infrastructure required to cover the entire household, as stated in [42].

Various non-wearable sensors are used in smart home environments: infrared motion, ultrasonic, photoelectric, video-based, vibration, pressure, magnetic switches, audio, and energy consumption sensors. However, sensors that provide rich information can be associated to a lower level of user perceived privacy (e.g., video and audio sensors), as discussed in [43].

The data collection procedure can be performed either directly from each IoT device to the cloud, or through an intermediate hub, usually stationary [44]. Another work proposed a hub resilient to cloud unavailability to provide critical functionalities to the smart home, and deployed in a Raspberry Pi device.

The ambient intelligence paradigm states that people can be assisted by learning and responding to their behaviour through technology integrated in their environment [43]. However, the deployment of machine learning models in smart environments face the additional challenge of resource constrained nodes; hence, solely considering performance-based metrics does not cover the entire problem. Thus, a method for considering trade-off between resource usage and performance in a machine learning hyperparameter tuning procedure is present in the literature [45].

Edge computing aims to bring computation closer to where it is needed. Its decentralized topology could be complementary to cloud computing, by dealing with the latter limitations (e.g., edge computing could reduce the latency of requests) [37,45]. Privacy by design principles are applied to systems design in order to mitigate privacy concerns at an early stage [46]. The Snips Voice Platform, as presented in [47], guarantees privacy by design (no user data is collected), with edge computing architecture (i.e., offline execution in a constrained node). Contrary to commercial voice assistants, it is cloud independent [12,47].

Machine learning algorithms applied to data that were collected from wearable and unobtrusive sensors were used for user behaviour identification in the healthcare domain. In this work found in literature, data was stored both locally and in the cloud, and home automation sensors were used in order to obtain information regarding the user environment. The authors highlight the possibility of generating alerts when user activities monitored by smart home sensors deviate from typical user behaviour [48].

VAuth is a continuous voice authentication solution with wearable sensors (e.g., eyeglasses, earphones, necklaces). An evaluation with 18 users and 30 voice commands demonstrated 97% person identification accuracy, with low latency [49].

Support Vector Machines (SVM), Random Forest (RF), and Naive Bayesian models were used for activity classification, and the RF model obtained the best results in the literature. One limitation is that the study was performed only in single resident smart home [50]. The Long Short-Term Memory (LSTM) model was used in order to predict user activities within a smart home for detection of anomalous user behaviour in [40]. A comparison over various Machine Learning algorithms for attack detection obtained the best accuracy for Decision Tree, Random Forest (RF), and Artificial Neural Network (ANN) [51].

An activity-aware approach for detecting anomalies based on activity monitoring was proposed in order to enhance smart home security. The security scenario studied was an invasion inside the smart home, and it used an isolation forest-based method to compute anomaly scores. Existing home security systems that are based on audio and video surveillance set up a possible risk of privacy for residents [34]. Most indicated authorization mechanisms for a smart home are based on attributes (e.g., actuator request timestamp in an Attribute Based Access Control—ABAC mechanism) [52]. A contextual-based framework for continuous authentication in smart homes is proposed in the literature as a way to enhance existing static user authentication mechanisms. The work highlights the possibility of using machine learning models in order to generate user profiles [22]. The proposed implicit authentication mechanisms for IoT environments can also be found in the literature: some are based only on smartphone data [22,53], while others rely on machine learning applied to WiFi signals [54,55] in order to authenticate IoT devices.

Considering multi residents in a smart home algorithm development is a common challenge that is found in the literature [42,50]. From the security perspective, Ref. [29] states that it is difficult to implement a granular access control inside multi-user environments with a smart speaker. A time clustering method for recognizing two-resident activities used open datasets from CASAS [41]. User labeling in multi-residential households could be performed through Bluetooth Low Energy wearable tags. Each tag could broadcast its unique identity periodically, and fixed scanners that are installed in each room could collect user indoor location data. However, a great challenge is to make users not forget to carry the tag [56].

The literature review indicates that securing IoT environments is still an open question. Constrained devices and privacy integrate some challenges in the field. Regarding smart homes, data collection methods, and even entire datasets can be found easily, and there is a wide range of work related to daily activity research. Security mechanisms are used in order to detect anomalies, or they rely primary on voice authentication.

The novelty of this paper is related to an non-invasive authentication for voice triggered transactions in smart home environments, which leverages behavior prediction. Its challenge response authentication protocol is designed to be suitable for constrained devices. The data collection procedure uses infrared and photoelectric-like sensors, which are classified as the most privacy-preserving ones in literature [43]. Our datalogger also differs by allowing local data storage in stationery hub, and the possibility of mobile and stationary intermediaries (e.g., smartphone and local computer, respectively), when considering an architecture that is inspired by the ZebraNet system [57].

As far as the authors are concerned, this is the first work to address voice triggered financial transactions when considering usable security, multi-factor authentication, and ambient intelligence paradigms.

## 3. Proposed Non-Invasive Authorization in Open Architecture

Two qualitative aspects are analyzed: how invasive the authorization method is, and whether the supporting IoT architecture is closed (i.e., consisting of a smart speaker solution vendor lock-in). The evaluation criteria are response time and security level of authorization approaches. The proposed scenarios were modeled based on existing authorization procedures. The architecture and interactions models are a simplified version of the architectures that are presented in [12,29].

**Figure 1:**
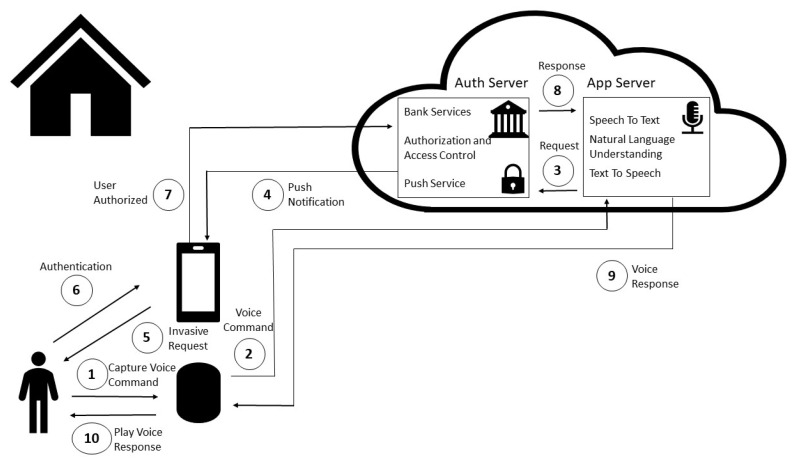
Closed Smart Speaker Architecture with Invasive Authorization based on push notification for user authentication through smartphone.

Invasive in Closed Architecture: invasive authorization based on smartphone notification and authentication procedure (e.g., facial recognition, fingerprint, voice, facial recognition, or password), in a closed smart speaker architecture is depicted in Figure 1.

First, the user commands the financial transaction by voice in a smart home environment (e.g., “Alexa, send 20 dollars to Fabio”) (1). The smart speaker inside the smart home environment captures the audio and relays it to the cloud services through the Internet (2). After Speech To Text and Natural Language Understanding steps in the cloud, a financial transaction intent is recognized, and an authorization request is issued from the application server to the authorization server (3).

The authorization server requests user authentication through push notification service (4). The user receives the request through a smartphone (5). Therefore, authentication is performed through existing authentication mechanisms in the smartphone (e.g., facial recognition, fingerprint, voice, facial recognition or password) (6).

The user authentication result is received by the authorization server (7) and, if the user is authorized to perform a specified action, the authorization server authorizes the transaction, thus triggering bank back-end services (bank services integration is beyond the scope of this paper). The result returns to the application server (8), the Natural Language Understanding module returns the success answer in text, and this text is converted into audio by the Text To Speech service, and then relayed to the smart speaker (9). Finally, the smart speaker receives answer audio and then plays it to the user in smart home environment (e.g., “Transaction successful”) (10).

**Figure 2:**
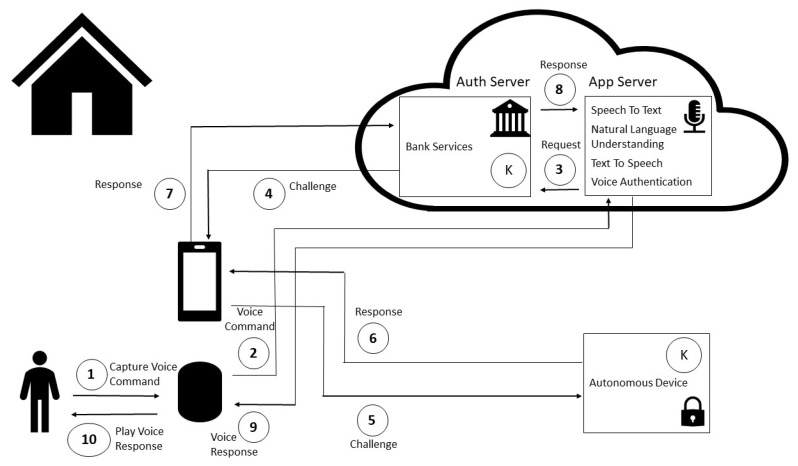
Open Smart Speaker Architecture with non-invasive Authorization that is based on data-driven approach.

Non-invasive in Open Architecture: implicit authorization through non-invasive authorization leveraging smart home behavior in an open smart speaker architecture is shown in Figure 2.

The user commands the financial transaction by voice in the smart home environment (e.g., “Alexa, send 20 dollars to Fabio”) (1); the Smart speaker captures the audio and relays it to the cloud through the Internet (2). In the cloud, the Speech To Text service processes the audio and then turns it into text. The Natural Language Understanding module) recognizes a financial transaction intent, and sends a request to bank server (3). The bank server issues a challenge to user’s mobile device (4), which relays it to an autonomous device in user’s smart home environment (5).

The specialized module performs machine learning algorithm inference that is based on latest smart home events to assess if the smart home behavior is occurring as expected. Behavior algorithm and smart home data collection procedures will be detailed in Section 4.2 and Section 4.3, respectively.

The autonomous device has a shared key (K) with the bank server, and uses it to send the encrypted response to the mobile device (6). Section 6.4 describes the enrollment scheme of shared keys. It is supposed that the smartphone has a local communication channel with the autonomous device (e.g., Bluetooth).

The mobile device relays the response to the bank server (7). The bank authorization server authorizes the transaction if the response to the challenge was correct, thus triggering bank back-end services for financial transaction fulfillment. The result returns to the application server (8). The text response is converted into audio by the Text To Speech service, and is then sent to smart speaker (9). Finally, the smart speaker in the smart home environment receives the answer audio and plays it to the user (e.g., “Transaction successful”) (10).

## 4. Behavior Learning Method

### 4.1. Synthetic Data: Coloured Petri Net

In this section, synthetic data used in initial stages of behavior learning for authentication purposes are described in detail.

Smart home sensor data are necessary for performing an exploratory analysis to assess the feasibility of detecting abnormal behaviour. However, it is not possible to know the ideal test bed setup prior to deployment [58], and the specificity of proposed scenarios makes it difficult to use existing smart home test bed data at first. Simulators have been used in order to deal with the difficulties of data collection in real smart home settings, with the benefit of facilitating the evaluation of algorithms in large amounts of data [5]. Therefore, a smart home data generator was constructed in order to overcome these initial challenges.

Contrary to existing model-based approaches, in which events or activities must be described in detail, the model used avatars in order to describe smart home residents, similar to interactive approaches to smart home data generation [58].

Another requirement is that the model must facilitate the generation of multiple occupancy datasets. Generating multiple user smart home datasets provides benefits not just to the present study, but also to a broad smart home research community [58].

Petri Nets are a consolidated method for graphical and mathematical notation for modeling and analysing a wide range of systems, including distributed systems [58,59]. In particular, Coloured Petri Nets (CPN) were used in order to develop the multi resident smart home data generator.

Standard Petri Net elements were used, as described: each resident is modeled as a token, the home rooms are places, and the events modeled in transitions occur when a person leaves one room and enters another.

The capability of Coloured Petri Nets to model timing behaviour was used to model the different “still timings” of different rooms (i.e., average time a person stays in a room after entering it). When a transition T is fired, the code fragment @+x adds a delay to the token [58] (i.e., after a person enters a room, its token is set to be active only after this delay). For different rooms, various “still timings” were set: five to Garage, Stairs, Storage and Hall rooms; 10 to Bath; 20 to Kitchen and LRoom; 50 to Laundry, RoomB, RoomG and RoomC; and 500 to Out.

The transitions between rooms were modeled according to each person. After a person enters a room, it only becomes active after the specified timing delay. Subsequently, the probability follows Table 1: in the first column, the first room denotes the room the person is in (i.e., previous room), and the following columns are the 4 possible persons modeled. For example, according to second column of Table 1, if Brother is in Hall, then he can go to RoomB (his room) with probability 0.5, to RoomG with probability 0.1, to RoomC with probability 0.1, to Bath (i.e., bathroom) with probability 0.2, or to kitchen with probability 0.1.

An executable Coloured Petri Net model was constructed while using CPN Tools [60]. By combining Petri Net modeling with high-level functional programming language (CPN ML [58]), the aforementioned time delays and different person probabilities could be described.

The colour set PERSON is timed in order to account for delays. It determines the types of tokens the places can accept [60] (i.e., “brother”, “sister”, “father”, “mother”) and it is associated to variable p, used in CPN ML functions integrated in the model. Different time delays were associated to each place.

Random variations were generated using pre-built ran() function, and the room transitions from Table 1 were modeled in functions written in CPN ML. The trans() function maps each probability to person p, and accepts probabilities according to the order (brother, sister, father, mother).

As the guard is a Boolean expression that must be True to enable the transition in a coloured petri net [60], the transition functions were used to compare present random generated number from 1 to 100 to the probabilities, according to each person p. For example, if "mother" is in Garage, then the random generated r in a 1 to 100 range is compared to 90 and 10 limits, according to gar2out() and gar2sta() functions.

**Figure 3:**
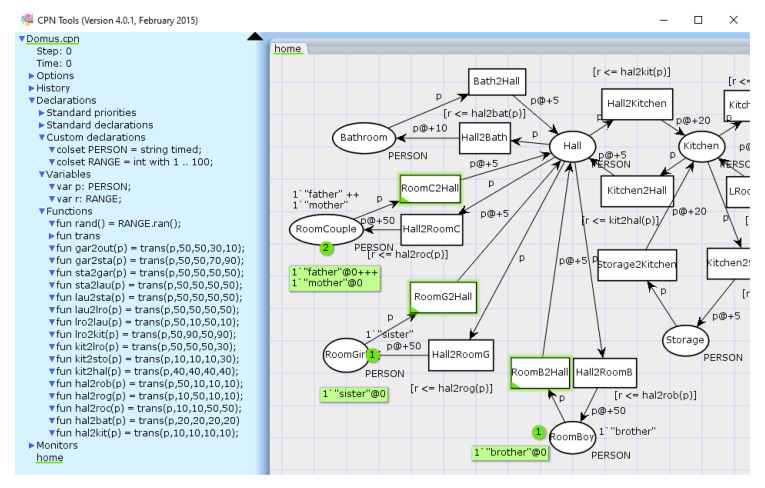
Coloured Petri Nets (CPN) ML declarations in CPN Tools for multi-user smart home data generator.

Figure 3 shows a screenshot of some of these functions in CPN Tools.

**Figure 4:**
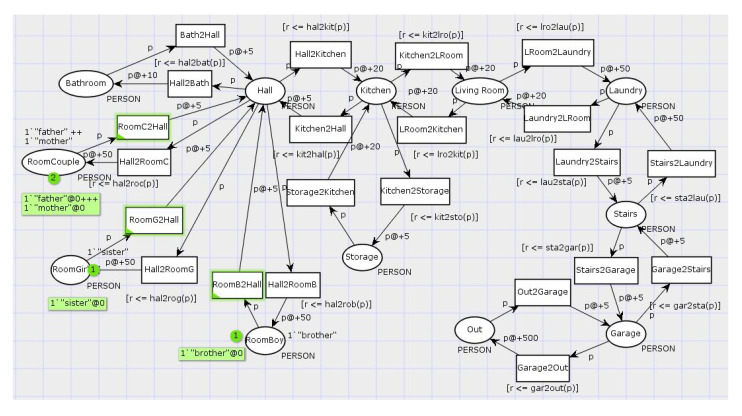
Executable coloured petri net model constructed in CPN Tools for smart home multi-user data generator.

Room transitions with person labels were collected while using CPN Tools monitor, specifically the data collector [60]. The log files obtained were parsed through simple Python script and put into csv format for exploratory analysis using machine learning algorithms. Figure 4 depicts the complete CPN model for synthetic data generation. The model was based on the test bed topology (described in the next section) and existing personas to facilitate further comparison between real and simulated scenarios.

### 4.2. Real Data: Smart Home Test Bed

While synthetic data generation is of foremost importance to smart home algorithm developments, having real open data sets that could be used as a benchmark is equally important [5].

The infrastructure used for data collection is based on the cumulative result of a four-year smart home project. Hedwig smart home architecture that was developed in 2017 focused on fault tolerance. The architecture was used for constructing a Brazilian smart home test bed in 2018, with data collected throughout 2019. In 2020, smart home modules were integrated to the OKIoT (Open Knowledge IoT Project) [61].

**Figure 5:**
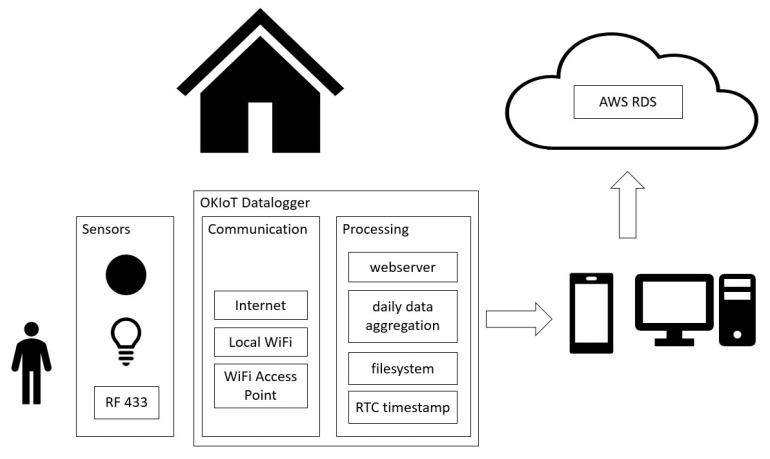
Test bed data acquisition infrastructure. Presence and room light switch events are recorded by the data logger, aggregated, and then sent to cloud through intermediary device.

Figure 5 illustrates the data acquisition procedure. Arduino-based sensors are responsible for environment monitoring and event broadcast. Movement and light bulb/switch state change triggers Passive InfraRed (PIR) and light bulb sensors in each room of the smart home test bed. These sensors broadcast each event through a different Radio Frequency code at 433 MHz (RF 433).

Events are captured by data logger through RF 433. The data logger includes a timestamp that is based on integrated Real Time Clock (RTC), and then stores it in the filesystem with daily granularity (i.e., events are stored in different files according to their timestamp to facilitate file management in the constrained device).

Aggregated data are made available through HTTP requests handled by data logger webserver. Three-level fault tolerant architecture [62] makes the data acquisition tolerant to momentary communication unavailability: if there is no Internet connection at the smart home, intermediary devices could connect to the data logger through the local WiFi network. If the local WiFi network is unavailable, the data logger module activates its Access Point, and the data can be acquired with a direct connection between the data logger and the intermediary device.

The intermediary device could be a smartphone that accesses the data logger web interface and gathers the data, or even a local computer. As the data logger can store up to 1 month of sensor data, a daily data acquisition routine was automated with a python script in a local computer in the test bed. This script obtains the data that were organized by day and sends them to a AWS RDS (Amazon Web Services Relational Database Service) with a MySQL database instance. The data logger is available in Public GIT repository, and a mobile app that automates the data acquisition procedure is subject to future developments.

**Figure 6:**
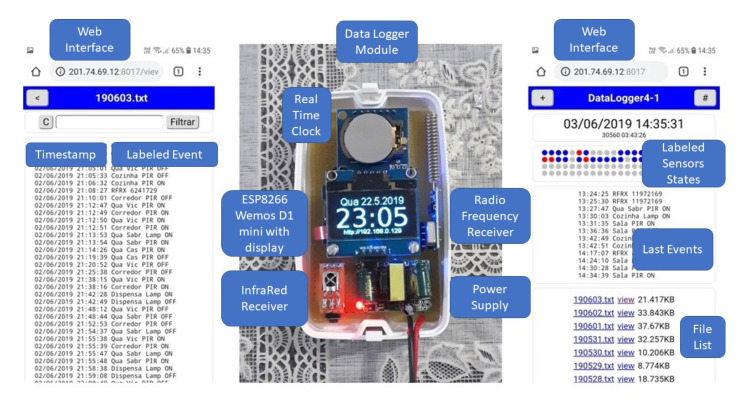
Data Logger module with Real Time Clock, compatible with Radio Frequency (RF433) signals, WiFi connectivity (Access Point and Client) with 3MB file system based on ESP8266 IoT development board. The log capability in the Brazilian smart home test bed with 20 sensors and four residents was of one month.

The OKIoT Data Logger is a data storage module compatible with Radio Frequency (RF433) signals. It is based on the ESP8266 IoT development board. Through RTC (Real Time Clock) and internal flash memory, the module record data sent by Arduino-based sensors, with the benefit of not needing additional external memory module (e.g., SD card, flash, or EEPROM memories). Its integrated battery makes it resilient to power outage events, and the file system has a capacity of 1.5 MB and 50 files for event data storage. Figure 6 depicts the Data Logger module.

The left part of Figure 6 depicts the web interface for a log of one day (specifically, 3 March 2019), accessible through smartphone by direct connection to the module, or by the local WiFi network. It is possible to create custom labels for RF 433 codes, which could help to identify the sensor and the room the sensor is installed. Even though the data is recorded in UNIX timestamp, the web interface displays it in an easily readable format, and also provides a filtering functionality. On the right part of Figure 6, a visualization for the current home state, last previous events, and an index of available files with data aggregated by day is presented. This interface was used for data collection setup and calibration in the smart home test bed.

In the central part of Figure 6, the data logger hardware module that is developed and installed in a real household is presented. Its display shows present timestamp, and the local IP address to connect to the module in the local network. With update-over-the-air feature and routines to discard old files to manage the limit of 50 files of ESP8266, calibration was made for one month.

Arduino-based sensors with Radio Frequency (433 MHz) transmitters were developed and installed in a smart home test bed in Brazil in 2018. Each room was equipped with a PIR (Passive Infrared) and main light state sensors in order to capture movement and lighting events (i.e., turning the light switch on and off). The objective was to be as the least intrusive possible, so that the daily routine of residents would not be greatly impacted. The total deployed sensors is of 20, two in each room: one PIR sensor and one main light state sensor.

**Figure 7:**
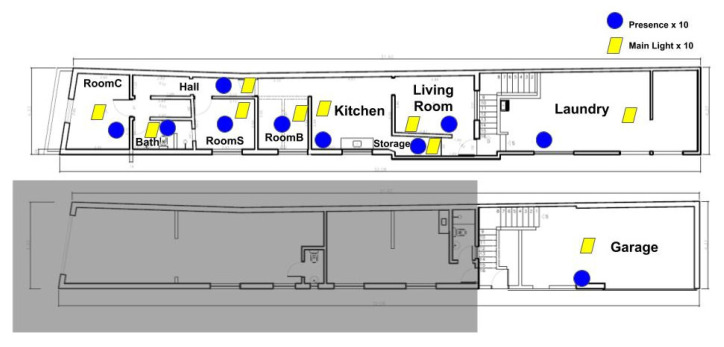
Layout and sensor setup of smart home test bed in Brazil. The household has two floors, and most of the house is in the upper floor. The shadowed area on the ground floor is not part of the household.

Figure 7 illustrates the sensor placement inside the Brazilian household. Each room has an equivalent room in CPN model from Figure 4. The main differences are “Stairs” and “Out” rooms: sensors placed in stairs faced interference, so the resulting data was not used; “out” room was included in CPN to model people absence from home, but it is not an actual room in the test bed.

Three open datasets found in the literature [63,64,65] were compared based on four criteria: state and event modeling, if data were aggregated or not, and if they provided description of the household and its residents, as portrayed in Table 2. State and event modeling aspects are based on the model the data is registered: series of events over time or periodic state-based. Available aggregated data can make their processing easier, and a detailed description of data is important in order to understand the context of the household.

Among the available smart home datasets, the CASAS dataset was chosen because of its detailed description, which will support further discussion and analysis. CASAS dataset #6 [63] has two residents, 51 sensors, and data were collected for one week. CASAS dataset #7 [66] has two residents, 71 sensors, and data were collected for two months, as summarized in Table 3. The idea is to perform an exploratory analysis in a multi-user scenario and investigate the applicability of the algorithms developed in other households with shorter and longer data collection periods.

### 4.3. Machine Learning

Supervised machine learning algorithms were developed for single user and multi-user scenarios. Synthetic data that were generated with CPN Tools, real data collected in the test bed, and open smart home data from CASAS were used to train and test the models. The algorithms were evaluated based on accuracy and Mean Squared Error (MSE) metrics.

Single user classification was performed with random forest algorithm. Single user context information was derived while using last 20 previous events by room.

The prediction of events for the whole household was performed in a multi-user scenario using the Long Short Term Memory model, based on the Tensorflow and Keras Python libraries. LSTM for time series prediction was deployed in a Raspberry Pi 3 for response time evaluation.

## 5. Behavior Algorithms Results

### 5.1. Single User Prediction based on Synthetic Data

The objective considered was to develop algorithms that could provide indoor context information in order to support context-based authorization rules. The algorithm is based on the count by room of the 20 previous events, and therefore predicts the probability that the person is in a certain room. Random Forest algorithm was selected, and hyperparameter tuning was performed, based on weighted accuracy metric when considering all classes (number of trees n from 1 to 100). Training and validation sets were composed of 70% and 30% of total events, respectively.

Table 4 summarizes the results. Even though weighted accuracy for all rooms is low (around 60% for sister and brother personas), the algorithm could predict whether or not each persona was at home or not (out class), with more than 90% accuracy.

The possibility to know with more than 90% accuracy that a certain person is at home by leveraging 20 previous smart home events is important to the the proposed authentication scheme. The trusted location will be discussed in Section 6.4.

### 5.2. Multi-User Scenario Prediction Based on Real Data

The prediction of room events for the whole household was used to handle the challenge of a multi-user smart home scenario, wherein the events are generated by more than one resident. The idea is to predict events based on historical events data, and compare with occurring events. Normal situations could be assessed by making sure that ongoing events in the smart home occur according to expected home behaviour.

The algorithm used was Long Short Term Memory (LSTM), because of its suitability to handle time-series data, and the metric used for comparison was the mean squared error (MSE). Real data were obtained from the OKIoT test bed (3370 events from one-month data), and synthetic data from CPN Tools model (with four residents, 4304 events in total). Each room was labeled with one number ranging from one to nine in the data preparation step. The training and validation sets were composed of 70% and 30% of total events, respectively. The window considered during the training was of 20 events. Tensorflow was used in Google Colab notebooks inside a cloud computing environment.

The LSTM model performed with MSE lower than 3 when frequent event sets contain seven events or less. Different number of frequent events were used to test the limitation of the model developed. As the events are concentrated on most frequent events, it was expected that, for this test bed, specifically, predicting a strict set of most frequent events (sseven most frequent events) could be easier than predicting all the possible events within the smart home. It holds true for both synthetic and real data.

When considering smart home data from the test bed, the lowest MSE was obtained with a window size of 20 events. The hyperparameter was experimentally obtained by varying the window size for the LSTM model from 1 to 100. The hyperparameter tuning is critical for optimizing the machine learning algorithm performance, and it is used in the model deployment in different devices in Section 5.3.

The same method for best window size parameter was applied to two open smart home datasets found in the literature, as portrayed in Table 3. For the dataset with one week of data (CASAS smart home test bed #6), the MSE results point unfeasible accurate smart home event prediction, even with 51 events considered. CASAS dataset #7 contains two months of data, with 71 sensors considered. For this dataset, the lowest MSE result could be obtained for a windowsize of 50. Both of the datasets had only two residents.

Exploratory developments in smart home test beds with 2 residents, but including pets (e.g., CASAS datasets #14 and #16) showed high values of MSE, showcasing that these scenarios need further developments.

### 5.3. Cloud vs Local Time Evaluation for Prediction Using Test bed Data

The developed LSTM model for event prediction in a smart home is considered as a behavior predictor. It is supposed that the comparison of real time events with predicted events can indicate the adherence of occurring behavior to expected behavior. For example, if there is an abnormal behaviour inside the smart home, then the authorization can be blocked, as it raises suspicion of malicious activities by the autonomous module. Please refer to Section 6.4 for behavior enrollment description.

A performance analysis was conducted for the LSTM model, with the OKIoT test bed data of one month in order to evaluate the feasibility of behavior prediction on the edge. The most frequent events were considered (a total of 10), window size of 20 events (based on the results from Section 5.2), with 70% and 30% of total events for training and validation. The model was deployed in three environments: cloud, local computer, and Raspberry Pi 3 module. The main framework used was Google Tensorflow with Keras library.

As depicted in Table 5, the results in the cloud and local computer were similar, but the model that was deployed in constrained IoT module showed an estimated inference time of 0.2 s, mean squared error four times greater than other alternatives, and greater training time (700 s against 20 s). Based on these results, training could be performed in a local computer or in the cloud, and inference could be performed in constrained IoT module. Even though the Raspberry Pi estimated inference time is of 200 ms against 10 ms of other alternatives (20 times greater), the perceived response time from a human standpoint is not much impacted.

**Figure 8:**
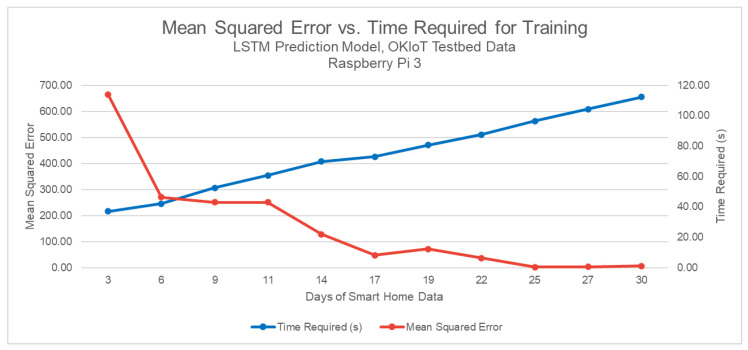
LSTM model with OKIoT test bed data deployed in Raspberry Pi 3 module in an edge computing architecture.

The graph presented in Figure 8 depicts how the mean squared error and time required for LSTM model training vary in increasing total events (OKIoT data of three days to 30 days was used). The trade-off between time and accuracy is clear: with fewer days in the dataset, the model is trained and validated fast, but the MSE is high. With all days in the events dataset, MSE is low, but the time that is needed for model training is high. With 2770 total events, or 25 days of smart home events, MSE gets to a minimum. Thus, including further days only hinders performance, as the time needed grows, but with no substantial improvement in accuracy metric is yielded. Hardware dependence is a negative characteristic of Artificial Neural Networks highlighted in a comparative study for human identification [67].

### 5.4. Open|Closed Architecture and Invasive|Non-Invasive Authorization

The final response time gains from open architecture with non-invasive authorization procedure, based on the LSTM model developed for the OKIoT test bed is estimated in this section.

The response time for each smart speaker basic services was obtained in a previous work. An API was developed for each basic smart speaker service: Speech to Text, Natural Language Understanding, and Text to Speech. The API supports two providers for each basic service, so one can evaluate the possible combinations of smart speaker services. The API was run on Dell Inspiron notebook with 16 GB RAM, 7th genaration Core i7 processor, Windows 10 operating system, connected via Ethernet to 60 mb Internet connection. Audio recordings in Brazilian Portuguese were analyzed, with a total of 200 tests performed. For more detailed description of response time results, please refer to [61].

**Figure 9:**
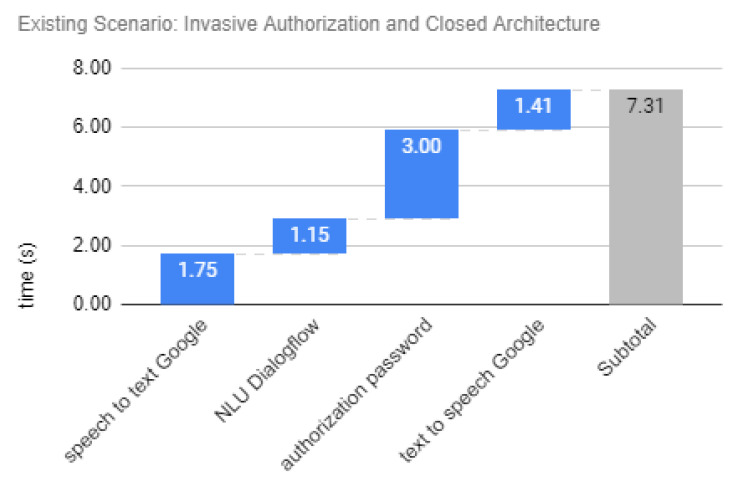
Total response time in the existing scenario: closed architecture and invasive authorization.

Existing scenario response time is represented in Figure 9, an example of response time that is required by use case performed in the architecture depicted in Figure 1. Closed architecture configures a vendor lock-in (i.e., only smart speaker services of one vendor could be used), and invasive authorization hinders usability by requiring a password based on push notification in smartphone.

The quantitative results from a previous work [61], and an user response time of 3 s for password-based authorization were considered for analysis. Thus, a smart speaker from Google while using a bank-related application for financial transaction through voice interface would have an estimated total response time of 7.3 s. The basic services response times considered are similar to the results of the response time evaluation over public cloud voice services found in [68]: 2.2 s for STT, 1.2 s for NLU, and 1.3 s for TTS.

**Figure 10:**
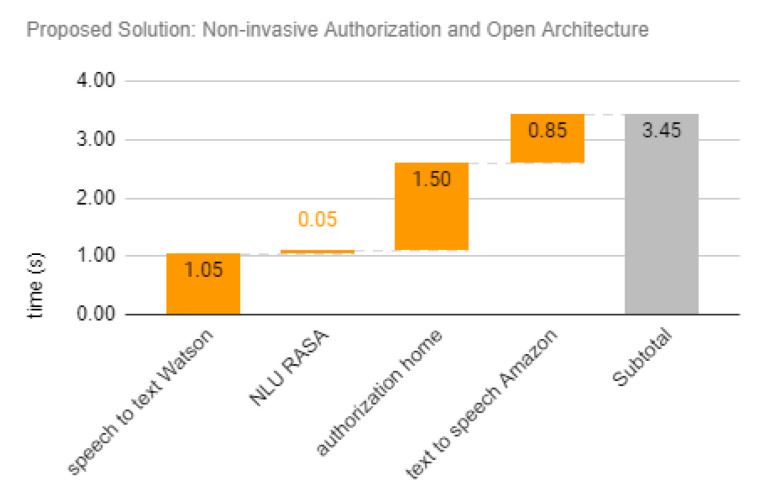
Total response time in the proposed solution: open architecture and non-invasive authorization.

The proposed solution is based on open architecture (i.e., smart speaker could choose basic services from different providers) and local non-invasive authorization procedure based on developed LSTM event predictor. After speech to text procedure, a natural language understanding process could be executed in the edge for privacy and performance reasons, as shown in Figure 1. With estimated inference time of 0.2 s in a Raspberry Pi 3 device, 1.3 s for local communication, and non-invasive authorization procedure could be performed in 1.5 s. Finally, with the faster Amazon text to speech, the total response time of the proposed solution is estimated at 3.5 s. The NLU response time of 0.05 s considered is similar to the Raspberry Pi 3 NLU response time result of Snips, of 0.06 s [47]. Figure 10 shows the resulting total response time.

To analyze the effect of invasive and closed architecture aspects in total response time, four scenarios were considered:

- closed and invasive: existing authorization mechanism based on smartphone notification with a single smart speaker cloud services provider (Google);
- open: open smart speaker architecture allows local NLU service (RASA), and the combination of Natural Language services from different providers (IBM for Speech To Text, and Amazon for Text To Speech), but the authorization is still invasive;
- non-invasive: transparent authorization based on autonomous module enables friction-less financial transaction, but smart speaker services are still all cloud-based, from a single provider (Google); and,
- open and non-invasive: combination of open architecture and non-invasive authorization from previous scenarios, with Natural Language Understanding and Authorization performed in the edge.

From the existing closed architecture with invasive authorization mechanism of 7.3 s, the open architecture could save 2.4 s, or 32% of the total response time by using the local natural language understanding module, as portrayed in Table 6. The non-invasive authorization mechanism that is based on LSTM deployed in autonomous module deployed on edge could save 1.5 s, or 21% of the total response time by not requiring user password for low value financial transactions. By combining both solutions in an open architecture with non-invasive authorization solution, the total response time could be halved, from the existing 7.3 s to 3.5 s.

## 6. Autonomous Device Challenge Response Authentication

In this section, the autonomous device and proposed authentication protocol are described in detail. The considered system model with relevant entities is presented. Security concepts, requirements and definitions support the specification of enrollment and protocol for an autonomous device used in proposed non-invasive authentication. Security considerations regarding some attacks close the section.

### 6.1. System Model

**Figure 11:**
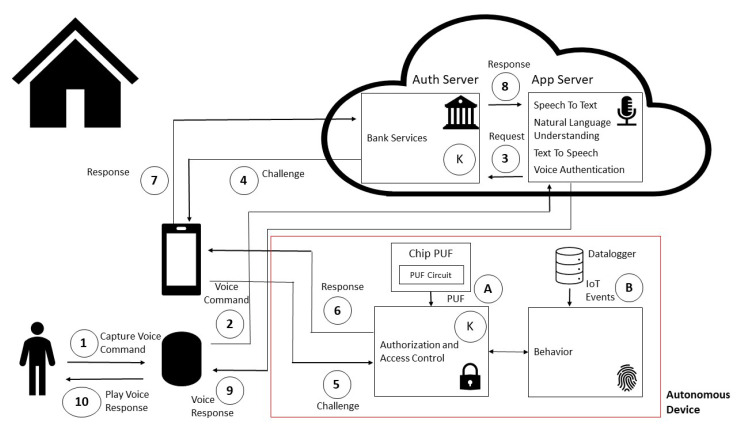
Proposed non-invasive authentication protocol with autonomous device in detail.

Figure 11 depicts a detailed view of the proposed solution. Suppose that user Victor wishes to transfer 10 dollars to user Fabio, and issues voice command through a smart speaker device in a smart home environment.

Victor issues voice command “Send 10 dollars to Fabio” (1). The audio is captured through a smart speaker and sent to the cloud (2). Cloud Speech To Text service transforms sound to text, and the Natural Language Understanding module recognizes an intent of “money transfer”, value of “10 dollars”, and a “sendTo” slot “Fabio” (person to send the money to). These cloud voice services also perform voice authentication in order to verify if command’s voice is really Victor’s. If the trust is high enough (parameters will be detailed further in the enrollment phase description), then a request is sent to bank server (3).

The bank server issues a challenge, which is sent to Victor’s mobile device (4), and is relayed to the autonomous device in Victor’s smart home (5). The communication is performed via Bluetooth, with an authentication protocol further detailed.

Smart home lightning and motion events are continuously captured through data logger module via Radio Frequency communication (433 MHz), which send the data to behavior module. The random forest and LSTM algorithms provide trust indicators of Victor being at home, and the adherence of home behavior with expected conduct (B). If the behavior is occurring as expected given a specified threshold, the autonomous device answers the challenge. This answer is encrypted using a common shared secret between the autonomous device and bank server (6). The response is relayed to the bank server (7).

If the authentication was successful, the bank server executes financial transaction fulfillment, effectively sending 10 dollars from Victor’s account to Fabio’s bank account. The result is sent from authorization and access control module to the edge voice services module (8). The cloud text to speech service converts text response into audio, which is sent to smart speaker device (9). The voice response is played in Victor’s smart home environment to the end user (10).

Additionally, a Physical Unclonable Function (PUF) is used as a source of randomness in random number generation for challenge creation by autonomous module (B) in order to provide mutual authentication between bank server and autonomous device. Therefore, the proposed scheme uses a combination of shared secret (K) for symmetric cryptography and user behavior in a trusted location (smart home). The shared secret (K) is associated with an unique autonomous device, which is associated with the user’s bank account. Section 6.2 provides more details about PUF.

The entities present in Figure 11 and considered in the proposed solution are described:

- Bank with support to voice financial transactions, with integrated bank server.
- Mobile device with communication with bank server and Bluetooth communication with the autonomous device.
- Smart speaker responsible for hands-free, voice-based interactions with end user.
- Cloud voice services with natural language understanding, speech to text, and text to speech services in end user’s language.
- Autonomous device, responsible for smart home events logging, authorization, access control. It has Bluetooth communication with the mobile device.
- End user who has smart speaker and autonomous device at home. The end user is also a client of the bank and has a personal mobile device and associated bank account.

### 6.2. Security Concepts

Physical Unclonable Functions (PUF) are mechanisms that can be used in challenge-response protocols as sources of randomness. By using their physical characteristics (PUFs can be silicon or non-silicon based), strong PUFs can generate large Challenge Response Pair (CRP) spaces [69,70]. Another role of PUFs in authentication protocols regards session key generation [71].

**Figure 12:**
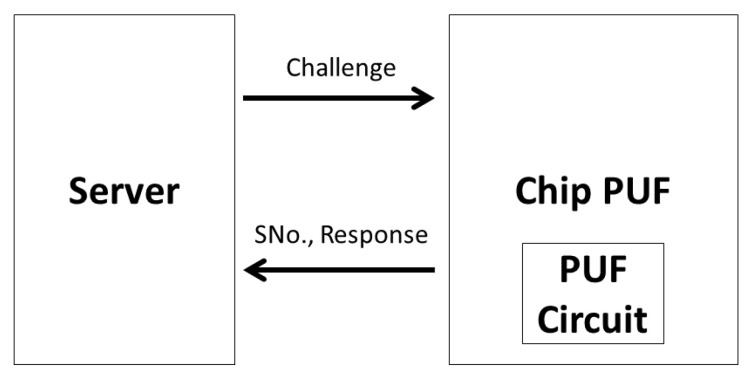
PUF based enrollment. Adapted from [20].

A PUF-based enrollment consists of an offline provisioning procedure wherein the PUF chip is directly connected to a fog/edge device (considered a server entity), as illustrated in Figure 12. A single random serial number is the id of the PUF device and is sent together with the response to challenges issued by server. The challenge response pairs (CRP) are mapped with the serial number and sent from server to the cloud in a secure manner.

A strong PUF can generate a high number of challenge response pairs and it can be used for identification and authentication purposes. When used directly in authentication, each challenge response pair must be used only once. Some examples of strong PUFs are based on delay characteristics (e.g., arbiter PUF, ring oscillator PUF) or in memory production process uniqueness (e.g., DRAM PUF) [69].

ODonnell et al. [72] proposed PUF-based random number generation, with promising results from NIST statistical test suit for randomness [73]. Maiti et al. [74] presented a true random number generator based on ring oscillator PUF, and Wortman et al. [70] proposed a consumer electronic device authentication scheme while using ring oscillator PUF as a seed for a pseudo random number generator. Therefore, a suitable PUF for proposed challenge-response scheme is the ring oscillator PUF, specifically for nonce generation, as further detailed in Section 6.5.

### 6.3. Security Requirements

Uniqueness is defined as the property in that the authentication of different entities must be different from each other. Timeliness requirement states that authentication requests must be unique in time and support transactions variant in time [75].

The proposed scheme must support mutual authentication between entities. When performing authentication between two entities, the procedure must be mutual, i.e., each entity must assure the identity of the other one. For example, the existing hand-held passcode generator is a lightweight scheme, but it does not support mutual authentication [75].

As the authentication protocol will be used to secure financial transactions, a critical requirement is that the proposed scheme must be strong enough to support online financial transactions, with a security level at least equal to the existing authentication methods that are used by banks [76,77].

It is common for security enhancements to hinder usability aspects. Existing authentication mechanisms, such as passwords, tokens, or two-stage PINs used in bank authentication are invasive and not appropriate to the usability of voice-based interactions. The use of usable security paradigm could help to design an authentication mechanism that is secure and usable in the user’s perspective [52,78,79]. Specifically, existing invasive authentication methods hinder the usability by requiring additional user interactions, as described in Definition 1.

**Definition** **1.** *The invasiveness of an authentication method is related to how many additional interactions an end user must perform to authenticate itself. In a voice-triggered financial transaction, the existing active interaction is the voice command issued once by end user, and captured by the smartphone or smart speaker device. Invasive procedures would be to ask for the user to provide a four-digit PIN, to enter a password in the smartphone, or even answer an additional question by voice. Facial recognition is an invasive authentication by this definition, because the user must make an additional interaction with the mobile device.*

**Definition** **2.** *A non-invasive authentication method is an authentication method, wherein the end user does not need to perform additional interactions to authenticate itself. In this case, the authentication procedure is executed in a transparent way to the end user, with no additional interactions, when considering the existing active voice interaction, as described in Definition 1. The non-invasiveness aspect of the solution herein defined regards additional interactions after the enrollment phase and it is not related to the privacy aspect.*

### 6.4. Enrollment Scheme

The enrollment scheme consists of user registration, associated with bank information and authentication rule for voice-triggered transactions, as presented in Definitions 6 and 7.

**Definition** **3.** *A trusted location TL of a user U is a location U visits with a frequency F, F being at least weekly. Some examples include home and work locations. One trusted location can be associated with more than one user. The enrollment of trusted locations can be performed when the end user is at the present location through its mobile device. The behavior that is related to specific trusted location is captured continuously by an autonomous device TD deployed at a trusted location. At first, the behavior is non-existent, but after some weeks upon TD deployment, TD can model the trusted location behavior based on the environmental data that were collected. For an example of smart home that can be used as a trusted location, refer to [61].*

**Definition** **4.** *The trusted autonomous device TD associated with a trusted location TL is a device that is associated with a user U that has a common shared secret K with bank server BS. One trusted autonomous device must be associated with one or more trusted locations (e.g., home and workplace). The random serial number that is generated by the PUF chip is used as an active device id; in the offline provisioning phase, a set of serial numbers related to a specific autonomous device can be registered; it enables further periodic update of the TD’s serial number. TD has only one active identifier, but BS can have multiple identifiers, which can be used by different autonomous devices, so, if a bank id is compromised, the negative effects are lessened. All of the mappings are stored in a secure secret database of bank server BS.*

**Definition** **5.** *Banking information BI associated with bank account BA of user U consists of*:

- Trusted location TL: as specified in Definition 3.
- Trusted device TD: as specified in Definition 4.
- Voice biometrics: voice model of user U for speaker identification.
- Password: static password, usually with 6 to 8 alphanumeric characters.

**Figure 13:**
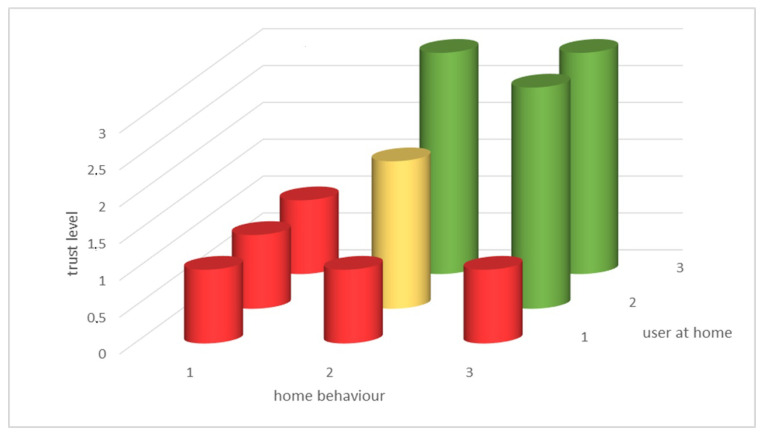
Trust level by location, device, and voice biometrics factors.

**Definition** **6.** *Trust level TLVL combines the level of actual, real-time trusted location behavior adherence to an expected behavior, and the level of confidence a specified user is at this trusted location. Figure 13 depicts an example of trust level. Low level of trust in home behavior or user at home models output represent low trust level.*

**Definition** **7.** *The authentication rule for voice-triggered transactions AR associated with user U consists of*:

- Transaction value: low, medium and high value transactions range must be specified by user.
- Trust Level TLVL as specified in Definition 6.

**Figure 14:**
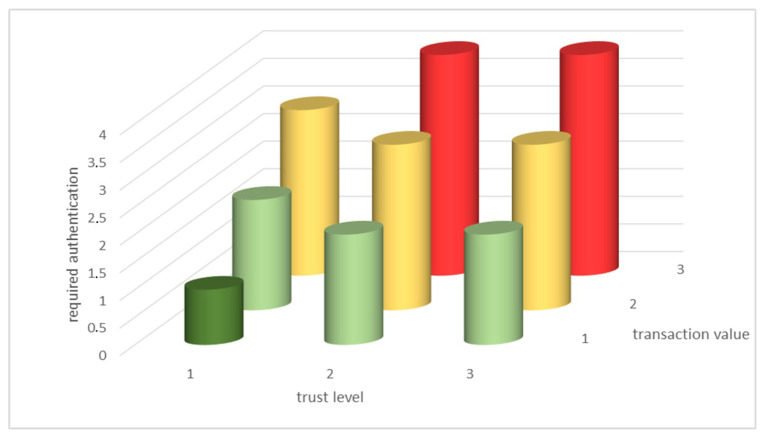
Required authentication procedures by the trust level and transaction value.

The required authentication procedure is related to the trust level and transaction value. If the transaction value is low, fewer authentication steps need to be considered, as the risk is considered to be lower. With a higher trust level TLVL, there are fewer authentication steps, as depicted in Figure 14.

### 6.5. Authentication Scheme

We assume shared key K between two entities that will perform mutual authentication. Entity BS is the bank server. Entity TD is the trusted autonomous device of the end user, which is located at a trusted location (i.e., bank client home, or workplace). The described authentication protocol must be performed after the authorization rule prescripts trusted device authentication, which will be performed in a challenge-response fashion.

**Definition** **8.** *(Adapted from [75]): a nonce is a value used only once, applied to prevent undetectable replay, usually implemented with random numbers, timestamps, or sequence numbers. The verifier in the challenge response protocol controls the time-variant parameter in order to provide timeliness and uniqueness. Uniqueness is required within a given time window (i.e., session).*

**Authentication Protocol** using SKID3 [80], referenced as a lightweight scheme in [75]:

1. Bank Server BS generates a random number *rBS* with sufficient randomness (possible due to high performance capabilities of bank server BS), which is used as a nonce to challenge the response protocol. BS sends random number *rBS* in clear text to autonomous device TD as the first challenge.
2. Trusted autonomous device TD receives random number *rBS* from entity BS. Autonomous device TD uses its Physical Unclonable Function (PUF) as a seed to a pseudo-random number generator in order to obtain random number *rTD*. With shared key *K* (unique for each autonomous device), random number received *rBS*, and random number generated *rTD*, entity TD uses a one-way (non-reversible) function *h* (the use of (keyed) one-way is a lightweight alternative to encryption algorithms):
  hK(rBS,rTD,bs)
  where: *rBS* is the random number generated by bank server BS; *rTD* is the random number generated by autonomous device TD; *bs* is the identifier of bank server BS; *K* is the shared secret between entities BS and TD; *h* is the one-way function (e.g., hash function); and, “,” denotes concatenation.
  Autonomous trusted device TD sends the result of one-way function as a response to the first challenge, and random number *rTD* that is generated to bank server BS as a second challenge.
3. Bank server BS receives one-way function computed by TD, and random number *rTD*. Entity BS performs look-up in its secret database to obtain shared key *K*. With *rTD*, shared key *K*, and *rBS* generated in step 1, bank server BS computes one-way function:
  hK(rBS,rTD,bs)
  where: *rBS* is the random number generated by bank server BS; *rTD* is the random number generated by autonomous device TD; *bs* is the identifier of bank server BS; *K* is the shared secret between entities BS and TD; *h* is the one-way function (e.g., hash function); and, “,” denotes concatenation.
  Entity BS compares the result of one-way function with received result from TD. If the values match, then the identity of autonomous device TD is verified by bank server BS, thus ending the first challenge.
  Bank server BS computes the response to second challenge by using one-way function:
  hK(rTD,rBS,td)
  where: *rBS* is the random number generated by bank server BS; *rTD* is the random number generated by autonomous device TD; *td* is the identifier of autonomous device TD; *K* is the shared secret between entities TD and BS; *h* is the one-way function (e.g., hash function); and, “,” denotes concatenation.
  Entity BS sends the generated response to entity TD.
4. Upon receival of response to second challenge, autonomous device TD computes expected response by using previous random numbers *rBS* and *rTD*, and shared key *K*:
  hK(rTD,rBS,td)
  where: *rBS* is the random number generated by bank server BS; *rTD* is the random number generated by autonomous device TD; *td* is the identifier of trusted device TD; *K* is the shared secret between entities BS and TD; *h* is the one-way function (e.g., hash function); and, “,” denotes concatenation.
  If the result that is received from BS matches the computed result, then the identity of bank server BS is verified by autonomous device TD.

The protocol messages are defined as:

TD ← BS: *rBS*
TD → BS: *rTD, hK(rBS,rTD,bs)*
TD ← BS: *hK(rTD,rBS,td)*

### 6.6. Security Formal Analysis Using BAN Logic

In this section, we present a formal analysis using Burrows–Abadi–Needham (BAN) logic on SKID3 protocol used in the authentication between bank server and trusted device. SKID3 protocol is indicated by Menezes et al. [75] to achieve mutual authentication for constrained devices. Bosselaers and Preneel [80] proposed the SKID3 challenge-response protocol based on ISO/IEC 9798-2 [81] with 64-bits challenges.

BAN logic is a simple logic that allows for the formal description of beliefs of entities involved in an authentication protocol, and the evolution of these beliefs [82]. Sierra et al. [83] considers BAN logic to be a simple yet powerful and robust tool for describing and validating authentication protocols, but highlighting its limitation to describe modern protocols where the value of postulates change during the run of the protocol. Sierra et al. [83] also consider BAN logic suitable to validate old protocols, so we consider it reasonable to use it to validate the SKID3 protocol.

Burrows et al. [82] formalism describes principals (e.g., P and Q), encryption keys (e.g., K), and statements. We summarize annotation, message-meaning, nonce-verification, formula components, and formula freshness rules that will be used in our formal security analysis. For detailed BAN logic formalism, please refer to [82].

Annotation rule: if the statement X holds true before message P → Q: Y then X holds true, and Q sees Y (Q ◃ Y) afterwards.

Message meaning rule for shared keys: if principal P believes that key *K* is shared with Q (P ∣≡ P $\overset{K}{↔}$ Q) and sees *X* encrypted under *K* (${\left\{X\right\}}_{K}$), then P believes that Q once said *X* (P ∣≡ Q ∣∼ *X*).

Nonce-verification rule: if principal P believes that statement *X* could have been uttered in the present (P ∣≡#(X)), and that Q once said *X* (P ∣≡ Q ∣∼ *X*), then P believes that Q believes *X* (P ∣≡ Q ∣≡ *X*). It states that principal P believes that Q said statement *X* recently (i.e., statement *X* is fresh).

Formula components rule: if a principal P sees a formula, then P also sees its components, given P knows necessary keys.

Formula freshness rule: if one part of a formula is fresh, then the entire formula is fresh.

The first step is to define the initial assumptions:

(**1**) TD ∣≡ TD $\overset{K}{↔}$ BS
(**2**) BS ∣≡ TD $\overset{K}{↔}$ BS
(**3**) TD ∣≡#(Ntd), where Ntd is the nonce generated by TD
(**4**) BS ∣≡#(Nbs), where Nbs is the nonce that is generated by BS

Assumptions (1) and (2) mean that TD and BS believe that they have a shared *K* key. Assumptions (3) and (4) mean that each principal believes in the random number freshness generated by itself.

The second step is to convert the SKID3 protocol into an idealized version with three messages M1, M2, and M3:

**(M1)** TD ← BS: *Nbs*
**(M2)** TD → BS: *Ntd*, ${\left\{(Nbs,Ntd,bs)\right\}}_{K}$
**(M1)** TD ← BS: ${\left\{(Ntd,Nbs,td)\right\}}_{K}$

The third step is to use BAN rules to derive beliefs from assumptions, based on messages specified in the idealized version of the protocol.

*TD receives M1*.

Apply annotation rule to (1), (2), (3) and (4) to obtain that these statements hold true after M1.

*BS receives M2*.

(**A**) Annotation rule with (4) yields: BS ∣≡#(Nbs)
(**B**) Annotation rule also yields: BS ◃ (*Ntd*, ${\left\{(Nbs,Ntd,bs)\right\}}_{K}$)
(**C**) Apply formula components rule in (B) in order to obtain: BS ◃ ${\left\{(Nbs,Ntd,bs)\right\}}_{K}$
(**D**) Message-meaning rule using (A) and (C) statements yields: BS ∣≡ TD ∣∼(Nbs,Ntd,bs)
(**E**) Formula freshness rule using (A) and (D) yields: BS ∣≡#((Nbs,Ntd,bs))
(**F**) Nonce-verification rule using (D) and (E) yields: BS ∣≡ TD ∣≡ (*Nbs,Ntd,bs*)
(**G**) Formula composition rule with (F) yields: BS ∣≡ TD ∣≡ *bs*

Statement (G) means that BS believes that TD has recently sent message with the *bs* identifier. Therefore, TD has authenticated itself with BS by providing BS identifier *bs*, based on shared key and hash function.

*TD receives M3*.

(**H**) Annotation rule with (3) yields: TD ∣≡#(Ntd)
(**I**) Annotation rule with (3) also yields: TD ◃ ${\left\{(Ntd,Nbs,td)\right\}}_{K}$
(**J**) Message-meaning rule with (H) and (I) yields: TD ∣≡ BS ∣∼ (*Ntd,Nbs,td*)
(**K**) Formula freshness rule while using (H) and (J) yields: TD ∣≡#((Ntd,Nbs,td))
(**L**) Nonce-verification rule using (J) and (K) yields: TD ∣≡ BS ∣≡ (Ntd,Nbs,td)
(**M**) Formula composition rule with (L) yields: TD ∣≡ BS ∣≡ *td*

Statement (M) means that TD believes BS has recently sent message with *td* identifier. Therefore, BS has authenticated itself with TD by providing TD identifier *td*, based on shared key and hash function.

The results support the mutual and freshness properties of indicated authentication protocol SKID3 to be used in our scheme. The reliance on identifiers confirms the claims that were described in the original SKID3 proposal [80].

### 6.7. Security Informal Analysis

**Definition** **9.** *(Adapted from [75]): impersonation is an attack wherein one entity (attacker) pretends to be another.*

An impersonation attack is possible if shared key *K* stored in the bank secret database is leaked; thus, securing the access to the secret database is a critical aspect of the proposed scheme.

A man-in-the-middle attack, wherein an adversary pretends to be entity BS to TD or vice-versa, can be mitigated with the proposed scheme, because of its mutual authentication characteristic. As stated in SKID3 authentication protocol original paper [80], an alternative would be to fool a legitimate entity. If an attacker were to act as a relay between BS and TD, then an impersonation would not be successful because of the names inserted into hash function in the protocol, which uses the shared key K unknown to the attacker.

However, in the previous scenario TD would respond to the attacker initial challenge. The authentication would not be successful, but we suggest that a penalty for failed attempts is integrated into TD: after a maximum threshold of failed authentication tries, TD could be blocked (e.g., not responding to any challenges), and it could be activated again by the legitimate user manually.

Another consideration regards the use of the smartphone device as a communication relay between BS and TD. One hypothesis considered in our proposal is that secure inter-app communications are assured by the operational system (i.e., communication between different apps installed in same mobile device). If this is not viable, then the mutual authentication protocol between BS and TD should be expanded to make TD only respond to an incoming challenge from trusted mobile app in order to mitigate inter-app attacks. The modified protocol could use a shared key between TD and smartphone. Such an expansion must consider different iOS and Android inter-app communication schemes [84], and a starting point could be the work of Elish et al. [85], who evaluated risk associated with inter-app communications in Android using static analysis, when considering not only how privileged operations are executed by single apps, but how apps with distributed privileges could be combined to perform malicious tasks (i.e., collusion attack).

**Definition** **10.** *(Adapted from [75]): replay is an attack wherein one entity uses information from previous protocol execution to deceive the same or a different verifier.*

The use of a challenge-response mechanism with nonce can prevent replay attacks, as stated in [75]. Each authentication attempt is protected by a unique fresh nonce (random number), used only once, which prevents replay attacks.

The formal security analysis in Section 6.6 confirmed the proposed authentication protocol resiliency against replay attacks. The proof was performed based on nonce freshness, initial hypothesis of shared key between Bank Server and Trusted Device, and the nonce-verification rule from BAN logic [82].

**Definition** **11.** *(Adapted from [75]): forced delay is an attack, wherein the attacker intercepts a message and relays it some instant later in time.*

The use of random numbers combined with short response time-outs is a principle to avoid forced delay attack. Random numbers are used in the proposed scheme; therefore, if a short response time-out is implemented, forced delay attack is prevented. A performance analysis must provide the expected delay ranges, which are the basis for protocol time out fine tuning.

**Definition** **12.** *(Adapted from [75]): chosen-text is an attack, wherein the attacker strategically selects challenges aiming at extracting information about the long-term key. It is possible in protocols which are not zero-knowledge based, because responses may reveal partial information about the claimant’s private key.*

A principle to avoid chosen-text attack is to embed in each challenge response a self-chosen random number. Random numbers *rBS* and *rTD* are embedded in the proposed scheme, for bank server BS, and autonomous device TD, therefore mitigating chosen-text attacks.

**Definition** **13.** *(Adapted from [75]): guessing is an attack, wherein the attacker randomly guesses a legitimate entity secret, in a local, remote, or offline (non-interactive) manner. If the attack is local and there is a penalty for failed attempts, then fewer bits of security are necessary; if the system is online and a remote attack can be performed and there is no penalty for failed attempts, or if attacker computations can be carried out offline (in a way the adversary can confirm the probability of impersonation before interacting with online system), more bits of security are necessary. According to OWASP [86], for symmetric encryption, using AES the key size must be at least 128 bits (AES-128); and, NIST [87] considers to be AES-128 acceptable.*

By definition, a PUF output is random, unique, and unclonable. Because their physical characteristics are used to generate PUF outputs on-time, and the output is not stored inside the device, PUF are more secure against offline attacks [69]. When considering OWASP and NIST guidelines, 128 bits of common shared key *K* must be used in order to prevent guessing attacks.

## 7. Discussion

Some of the solutions for authentication methods with increased usability are based on wearables. Flautner et al. [88] proposes a wearable trusted device that monitors continued legitimate user possession by proximity sensor or biometric measurement. Farraro [89] proposes a wearable device as a security token, that within proximity of protected system allows the user to perform a reduced security authentication. Feng et al. [49] proposed VAuth, a continuous authentication scheme that is based on wearables, such as earphones and eyeglasses, to match body-surface vibrations to speech signal received. VAuth could achieve 97% detection accuracy with 18 real users, while our results show 90% indoor state accuracy with four synthetic users (i.e., whether the user is at home or not).

Another solutions rely on smartphone data instead of smart home data. Whaley and Somerville [90] proposes accelerometer data to gather information of the user while walking, and infer a characteristic gait, unique to the user. Ashibani and Mahmoud [22] could authenticate users based on network traffic patterns of accessed apps in a accuracy range of 79% to 83% for 10 real users. Musale et al. [53] achieved 97% accuracy using random forest algorithm for 12 real users. Anjomshoa et al. [91] proposed a continuous authentication scheme leveraging five social networking apps usage data from smartphone devices, and could verify genuine users up to 97% success ratio for six real users; however, the architecture is cloud-centric, and it would not be suitable for our proposed edge computing architecture.

We consider that wearable and smartphone alternatives are suitable in another trusted locations, such as the work space. However, based on the finding that users tend to forget to carry tags in a smart home [56], we consider that our proposal is more suitable for the considered home environment, as the user does not need to carry anything: the smartphone must to be close to the trusted device, but the user does not need to carry it in our scheme.

One can also consider an approach that leverages environmental data to continuous authentication. Shi et al. [55] developed a deep learning user authentication scheme in university office and apartment environments, and could achieve over 94% authentication accuracy for 11 real users based on WiFi signals. Ongun et al. [38] collected data from 15 IoT devices in a lab to classify 6 real users with 86% accuracy, and 5 real users with 97% accuracy.

Table 7 summarizes accuracy results comparison with continuous authentication solutions found in the literature.

Tahavori and Moazami [92] proposed a lightweight PUF-based end-to-end key agreement scheme between smart meter device and service provider in the smart grid environment. Formal security analysis was performed by AVISPA and scyther tools, and computational and communication costs proved that the scheme is suitable for resource constrained smart meter devices.

Pérez-Jiménez et al. [93] used magnetic PUF and pseudorandom number generator in order to generate keys to be used in lightweight encryption process based on simple XOR. Their method included simulations scenarios that were created in MATLAB and Simulink software tools, NIST test suit to evaluate randomness of key generators, and Arduino Nano board as a hardware platform.

Our scheme uses PUF to enhance randomness of mutual authentication protocol nonce that is generated by resource constrained device, and it is based on hash function instead of XOR function. However, as it relies on a shared key between bank server and trusted device, we consider that it is desirable to integrate such key agreement and key generation schemes while using PUFs. Tahavori and Moazami [92] and Pérez-Jiménez et al. [93] works could help the enrollment process using PUF, and our scheme could provide mutual authentication while using PUF to enhance nonce randomness in the protocol.

Basin et al. [94] used the scyther tool to perform security analysis in the ISO/IEC 9798 standard (SKID3 protocol was based on this standard), and found that the protocols described in the standard guarantee aliveness. To enhance protocol security, Basin et al. [94] suggests principles, such as positional tagging (e.g., message components must uniquely identify origin) and inclusion of identities and their roles (i.e., each message component should include information regarding all agents that are involved in protocol run).

Ziauddin and Martin [95] used AVISPA tool to execute formal security analysis in the ISO/IEC 9798 standard, and showed that tow-party protocols are secure, but there are possible attacks against mutual authentication protocols that involve a third party.

The SKID3 protocol used in the proposed scheme can be enhanced in order to satisfy more security properties, and we consider that the principles described by Basin et al. [94] are relevant to achieving this goal. Further, enhancements should also consider possible attacks regarding a third party, possible according to Ziauddin and Martin [95].

## 8. Final Considerations

An alternative solution to the invasive authorization method for financial transactions triggered by voice was proposed and evaluated in this paper. Based on smart home data that were collected with sensors with high perceived privacy, local authorization procedure could be locally performed for performance and security aspects. An open smart speaker architecture facilitates security mechanisms integration, and enhanced response time by natural language understanding performed in the edge.

The proposed event prediction with LSTM could be used in order to authorize low value financial transactions, or to support multi-factor authentication for higher value transactions in multi-user scenario. The random forest algorithm achieved 90% accuracy for four synthetic users, and it could be used for implementing context-based authorization rules (e.g., authorizing transactions if there is trust that a specific person is at home).

The Random Forest algorithm was evaluated whileusing synthetic data that were generated in CPN Tools, and event prediction based on LSTM was evaluated in real data from OKIoT smart home. The prediction algorithm was deployed in a Raspberry Pi 3, and a response time analysis was performed. The proposed prediction based approach could halve total response time for low value financial transactions, from 7.3 to 3.5 s in an open architecture with a non-invasive authentication mechanism.

An autonomous module for enabling non-invasive authentication for voice-triggered financial transactions was specified. Enrollment and authentication schemes were designed based on security requirements. Some considerations regarding impersonation, replay, forced delay, chosen-text, and guessing attacks integrated an informal security analysis. A formal security analysis using BAN logic proved the mutual and freshness properties of the proposed scheme. The proposal considered multi-factor authentication and usable security paradigms, and uses Physical Unclonable Functions as a source of randomness.

Our contributions are based on open access to CPN model for multi-user smart home data generation, developed supervised learning algorithms, Brazilian smart home dataset of three months, and specification of a non-intrusive authentication scheme for voice-triggered financial transactions.

The advantages of proposed system regard the reduced response time due to open smart speaker architecture, increased usability as the user does not need to carry extra devices or perform additional actions, and lightweight mutual authentication scheme. The disadvantages regard the need for specific stationary trusted device, and manual enrollment scheme that must support shared key agreement between the trusted device and bank server, which can be a burden for the end user.

As future work, the challenge-response protocol for mutual authentication may be modified in order to enhance security. For example, we could increase the security between trusted device and smartphone (third party) to prevent malicious apps attacks, such as inter-app collusion attacks. The feasibility of electronic PUF random number generator with specific and general purpose devices could be validated when considering randomness metrics. The integration of the proposed scheme with IoT devices, with associated performance metrics for assessing the feasibility of implementation with constrained devices, is also planned. Key generation and key agreement schemes that are based on PUF could enhance enrollment scheme security.

## Figures and Tables

**Table 1:** Room transition probability for each of the four persons modeled.

Room2Room|Person	Brother	Sister	Father	Mother
Hall2RoomB	0.5	0.1	0.1	0.1
Hall2RoomG	0.1	0.5	0.1	0.1
Hall2RoomC	0.1	0.1	0.5	0.5
Hall2Bath	0.2	0.2	0.2	0.2
Hall2Kitchen	0.1	0.1	0.1	0.1
RoomB2Hall	1	1	1	1
RoomG2Hall	1	1	1	1
RoomC2Hall	1	1	1	1
Bath2Hall	1	1	1	1
Storage2Kitchen	1	1	1	1
Kitchen2LRoom	0.5	0.5	0.5	0.3
Kitchen2Storage	0.1	0.1	0.1	0.3
Kitchen2Hall	0.4	0.4	0.4	0.4
Laundry2Stairs	0.5	0.5	0.5	0.5
Laundry2LRoom	0.5	0.5	0.5	0.5
LRoom2Laundry	0.5	0.1	0.5	0.1
LRoom2Kitchen	0.5	0.9	0.5	0.9
Stairs2Garage	0.5	0.5	0.5	0.5
Stairs2Laundry	0.5	0.5	0.5	0.5
Garage2Out	0.5	0.5	0.3	0.1
Garage2Stairs	0.5	0.5	0.7	0.9
Out2Garage	1	1	1	1

**Table 2:** Comparison of open datasets from smart home test beds.

Test Bed Dataset	Publication Date	Size	State	Event	Aggregated	Description
casas (dataset #7 [63])	2009	0.6 MB		x	x	x
aras [64]	2013	225 MB	x			
domus [65]	2013	536 MB	x	x		

**Table 3:** CASAS datasets with two residents used for multi-user scenario algorithm.

Dataset	#6 [63]	#7 [66]
Description	Daily life, 2008	Daily life, Spring 2009
#Residents	2	2
Last Updated	24/05/2010	08/07/2014
Size data (MB)	0.6	4.6
Initial	24/06/2008	02/02/2009
Final	01/07/2008	04/04/2009
Days	7	61
Events/Sensors	51	71
Measures	20952	137789

**Table 4:** Indoor user context information accuracy from the 20 previous events count by room, Random Forest classifier with n hyperparameter tuning.

Random Forest F1 Score	Brother	Father	Mother	Sister
n hyperparameter	93	60	53	73
all rooms	59%	77%	84%	63%
out	93%	93%	96%	90%
own room	70%	76%	67%	73%

**Table 5:** LSTM model with Open Knowledge IoT Project (OKIoT) test bed data. One month, nine frequent events, window size of 20 events, 2359 events for training, and 1011 events for validation.

Time (s)	Cloud	Local PC	Local IoT
Training	15.18	11.99	534.49
Validation	8.26	9.15	186.94
Total Time	23.44	21.14	721.43
MSE	0.06	0.06	1.10
mean error	0.25	0.25	1.05
estimated inference time	0.01	0.01	0.18

**Table 6:** Total response time comparison for proposed alternatives with open/closed architecture, and invasive/non-invasive authorization method.

	Closed and Invasive	Open	Non-Invasive	Open and Non-Invasive
total time	7.31	4.95	5.81	3.45
time saving (s)		2.36	1.50	3.86
time saving		32%	21%	53%

**Table 7:** Accuracy comparison with continuous authentication solutions found in the literature.

Solution	Based On	Accuracy	Number of Users
VAuth [49]	wearable data	97%	18
Ashibani and Mahmoud [22]	smartphone data	79%	10
Musale et al. [53]	smartphone data	97%	12
Anjomshoa et al. [91]	smartphone data	97%	6
Shi et al. [55]	WiFi data	94%	11
Ongun et al. [38]	WiFi data	97%	5
Proposed	IoT events data	90%	4
